# Genetic correlations of environmental sensitivity based on daily feed intake perturbations with economically important traits in a male pig line

**DOI:** 10.1186/s12711-025-01000-1

**Published:** 2025-10-02

**Authors:** Tomasi Tusingwiire, Carolina Garcia-Baccino, Bruno Ligonesche, Catherine Larzul, Zulma G. Vitezica

**Affiliations:** 1 GenPhySE, Université de Toulouse, INRAE, ENVT, 31326 Castanet-Tolosan, France; 2 SAS NUCLEUS, 35650 Le Rheu, France

## Abstract

**Background:**

Pigs in intensive production systems encounter various stressors that negatively impact their productivity and welfare. The primary aim of this study was to estimate the genetic correlations of the slope (indicator of sensitivity of the animals to environmental challenges) of the daily feed intake (DFI) across different environmental gradients (probability of the occurrence of a challenge on a given day) with growth, feed efficiency, carcass, and meat quality traits using a single-step reaction norm animal model (RNAM) in Piétrain pigs. In addition, genetic correlations of DFI (its total breeding value) with the same traits were also estimated. The probabilities of the occurrence of an unrecorded environmental challenge, inferred via a Gaussian mixture model, were taken as a reference and used in the genetic analysis as an environmental descriptor. Variance components were estimated via restricted maximum likelihood using the single-step genomic best linear unbiased prediction method, using a series of multivariate RNAM with two phenotypes (DFI and each of the traits of economic importance), with the probability of an unrecorded challenge on a given day included as an environmental descriptor for DFI only, because DFI is recorded daily but the other traits are not.

**Results:**

Genetic correlations of the slope of DFI were 0.15 with age at 100 kg, 0.04 with backfat thickness, − 0.29 with loin muscle thickness, 0.05 with feed conversion ratio, − 0.07 with lean meat percentage, − 0.13 with pH of the ham at 24 h postmortem, 0.06 with drip loss percentage, and 0.15 with boneless ham weight. Complementary results showed that genetic correlations of DFI with other economic traits varied across the environmental gradients.

**Conclusions:**

Estimates of genetic correlations of DFI with other traits of economic importance varied across the environmental gradients, especially for growth rate, which suggests the presence of genotype-by-environment interactions. The slope of DFI is an indicator of sensitivity of the animals to environmental challenges. Most traits of economic importance exhibited weak genetic correlations with the slope of DFI, indicating that selection for resilience based on the environmental sensitivity (slope of DFI) can be performed without adversely affecting these other traits. Our results demonstrate the feasibility of improving resilience through genetic selection.

**Supplementary Information:**

The online version contains supplementary material available at 10.1186/s12711-025-01000-1.

## Background

Pigs in production systems must cope with short-term and long-term stressful factors that affect their productivity and welfare [1]. These stressors are environmental factors that include management practices, pathogen load, interactions with other animals, extreme weather conditions, etc. [2–6]. All these factors can affect growth, reproduction, behavior, immunity, and meat quality [1]. Therefore, breeding for resilience is becoming increasingly important in pig production [7]. Highly resilient animals are expected to be minimally affected by perturbations, or rapidly return to the normal state they had before being exposed to perturbations [8]. In general, less-resilient animals may exhibit either radical reductions or a high day-to-day variation in daily feed intake (DFI), whereas resilient animals have stable DFI [7, 9]. Moreover, Nguyen-Ba et al. [10] noted that perturbations such as heat stress or sanitary challenges typically induce transient physiological responses in pigs, characterized by changes in DFI and body weight gain. This broader view of resilience, which includes an animal's response to various stressors over time, represents general resilience and differs from the narrower concept of disease resilience, which relates to a specific challenge [8].

Measuring general resilience can be difficult since detailed information on specific environmental factors, such as air quality or pathogen load, is often not available in commercial production conditions [4, 11]. Therefore, many studies have focused on one particular type of resilience (especially disease resilience) and have used experimental set-ups to identify underlying physiological mechanisms [12–14]. However, Colditz and Hine [8] suggested that phenotyping for resilience should be conducted during animals' exposure to stress events intrinsic to their management environment, incorporating novelty components, social interactions, and changes in human exposure patterns, rather than under experimentally imposed stressful conditions [8]. In this work, we address resilience without artificially inducing stressful conditions, so we consider the various challenges an animal may face in its daily life under commercial production conditions.

Reaction norm models are essential for understanding the genetic basis of the expression of a trait across an environmental gradient (EG). In the reaction norm animal model (RNAM), breeding values are divided into two components: an intercept and a slope [15, 16]. The breeding value of the intercept represents an individual's genetic merit at a standardized environmental level, whereas the slope measures how much an individual's phenotype fluctuates across a covariate that quantifies different environmental conditions (environmental sensitivity) [14, 16–20]. The ideal reaction norm of a production trait has a high level (intercept) and a flat slope [15].

Multitrait reaction norm models can extend this approach by simultaneously considering multiple traits and their interactions across different environments [21, 22]. Santana et al. [21] used a multitrait RNAM on beef cattle raised in tropical conditions and discovered that genetic correlations between traits across the EG showed that selection responses can differ significantly depending on the environment considered. Using the same approach, Windig et al. [22] investigated the genetic relationships between milk yield, health, and fertility traits in dairy cattle across different herd environments and reported that the genetic correlations between these traits varied substantially across the EG.

Incorporating genomic information into the RNAM can increase the precision of genetic parameter estimates and breeding values. Zhang et al. [23] and Song et al. [16] applied single-step genomic best linear unbiased prediction (ssGBLUP) [24, 25] using a reaction norm model [24, 25] and obtained higher prediction accuracy than the pedigree-based reaction norm model. To use RNAM, known environmental variables must be included as covariates. Recently, Garcia-Baccino et al. [9] presented a data-driven method to estimate the probability that, on a given day, an unrecorded environmental challenge occurred, via a Gaussian mixture model. The estimated probabilities of a high coefficient of variation (CV) of DFI, i.e. of the occurrence of a challenge, were used as an environmental descriptor in an RNAM to evaluate the genetic determinism of sensitivity to the environmental challenges [9]. The probabilities of a high CV capture all the naturally occurring environmental challenges experienced by animals in farms, as opposed to experimentally imposed challenges. These challenges may include management practices such as ear tagging, weighting, feeding station repairs, and moving animals between pens, as well as issues like feeding stations blockages, and illnesses [26]. Tusingwiire et al. [26] applied this approach to DFI data from pigs using a single-trait pedigree-based RNAM and found that the slope of DFI is heritable and can effectively be used as an indicator of sensitivity to environmental challenges.

Given that the slope of DFI across the EG can serve as an indicator of environmental sensitivity [7, 9, 10, 26] and a previous study on pigs [26] showed the feasibility of improving resilience through genetic selection, it is of interest to identify an approach to include environmental sensitivity into breeding goals. A crucial step to this end is assessing the genetic correlations between the slope of the DFI (an indicator of environmental sensitivity) and traits of economic importance. In terminal sire lines such as Piétrain, these traits primarily include growth, feed efficiency, carcass, and meat quality. The Piétrain breed is widely used as a terminal sire line in pig breeding programs due to its very high lean-to-fat ratio to improve carcass traits and production efficiency [27, 28]**.** This breed originated from the Belgian village of Piétrain and has spread worldwide since the 1960s [29].

Following the reaction norm approach framework presented by Tusingwiire et al. [26], the primary aim of this study was to estimate the genetic correlations of the slope of DFI across different EG with various growth, feed efficiency, carcass, and meat quality traits, using a single-step RNAM in Piétrain pigs. For completeness, we also estimated the genetic correlations of the same traits with DFI across the EG.

## Methods

The data used in this study were recorded and provided by SAS NUCLEUS pig breeding company (Le Rheu, France). Animal care and use committee approval was not obtained for this study because the data were obtained from an existing database.

### Animals and phenotypes

Data for this study were obtained from 74,727 Piétrain pigs over a period of 6 years (2017 through 2023). Among these animals, DFI data used in this study were collected from 4788 males over a 3-year period (2021–2023) from 74 batches, where a batch was defined as a group of animals of the same age and weight. Each batch consisted of 47 animals on average, ranging from 23 to 55. For each batch, the animals were housed in groups of 12 to 14 animals within each pen at approximately 30 kg of body weight (BW) and 10 weeks of age. Each pen was equipped with an automatic concentrate feeder (ACF), and each animal was fitted with a unique radio frequency identification tag. The animals were automatically identified by the ACF each time they approached the feeder and the amount of feed consumed was recorded, defining a “visit”. A 7-day “adaptation” period was required for the animals to get accustomed to the new environment and learn how to use the ACF. After the adaptation period, animals were recorded for an average of 64 days (ranging from 38 to 76 days), starting from approximately 30 kg to 100 kg BW. All pigs were fed ad libitum and had free access to water during the whole period. Records from the adaptation period and those that had technical errors (e.g. data recording and transfer problems) were removed from the analysis. A total of 6,219,930 visits were recorded across all animals, with an average of 20.4 visits per pig per day. For each animal and each day, feed intakes per visit were summed to obtain DFI (kg/day), resulting in a total of 304,826 DFI records across all animals. Since data were collected on growing animals, and DFI (and its variance) increases as an animal grows, the natural logarithm of the daily CV of DFI was used to quantify variability in DFI across animals on a given day within each batch.

In addition to DFI, the other traits analyzed were the age (in days) at which the animal reached 100 kg (AGE), backfat thickness at 100 kg (BFT), loin muscle thickness at 100 kg (LMT), feed conversion ratio (FCR), lean meat percentage (LMP), pH of the ham at 24 h postmortem (PH24), drip loss percentage (DLP), and boneless ham weight (BHW). All these traits were measured in females and males, except for FCR which was recorded only in males. Note that these traits were measured once in the lifetime of the animal, contrary to DFI which was measured daily.

For AGE, all pigs were weighed at the start (${W}_{initial}$, kg) and end (${W}_{final}$, kg) of the testing period. The AGE was then obtained as: $AGE= {\text{age}}_{initial} + \frac{(100 - {\text{W}}_{final})}{ADG}$, where ${\text{age}}_{initial}$ is the age (days) of an animal at the start of the testing period and ADG (kg/day) is the average daily gain of the animal. The ADG was computed as: $ADG= \frac{{W}_{final}- {W}_{initial}}{{\text{age}}_{final}- {\text{age}}_{initial}}$, where ${\text{age}}_{final}$ is the age of an animal at the end of the testing period.

Traits BFT and LMT were measured using real-time ultrasound devices at standardized anatomical locations between the 3rd and 4th last ribs, and 6 cm from the mid-dorsal line. For each trait, the mean of all ultrasonic measurements was calculated and subsequently adjusted to a 100 kg live weight as follows: $BFT= {\text{BFT}}_{test}+{\beta }_{BFT}*(100-{W}_{final})$, $LMT= {\text{LMT}}_{test}+{\beta }_{LMT}*(100-{W}_{final})$, where ${\text{BFT}}_{test}$ and ${\text{LMT}}_{test}$ are the backfat and loin muscle thickness measured at the testing, respectively; ${\beta }_{BFT}$ and ${\beta }_{LMT}$ are the regression slopes of BFT and LMT on live weight at the testing weight (${W}_{final}$, kg), respectively.

Trait FCR is the ratio of DFI to ADG. Trait LMP is a metric used in the French pig industry to assess the quality of carcasses and it is calculated as follows: $LMP= 60.12 - 0.487 G3 - 0.133 G4 + 0.111 M3 + 0.036 M4$ [30], where G3 is the minimum thickness of fat covering the *gluteus medius* muscle; G4 is the average fat thickness over the fourth lumbar vertebra; M3 is the minimum thickness of the muscle between the anterior end of the *gluteus medius* and the dorsal part of the medullary canal; and M4 is the average thickness of the muscle covering the fourth lumbar vertebrae. Trait PH24 was measured in the *semimembranosus* muscle of the ham 24 h postmortem using a calibrated pH meter. Trait DLP is a measure of the amount of water lost from meat during storage and is an indicator of meat quality. Meat samples were weighed immediately after slaughter, stored under a controlled environment for 72 h and then reweighed. The DLP was then calculated as the difference between the initial and final weights of the sample and expressed as a percentage. BHW was measured using ultrasound scanning technology with Autofom III™ (Frontmatec, Kolding, Denmark) [31]. The average performance and the numbers of animals and records used for analyses of these traits are presented in Table 1.

**Table 1:** Number of animals, records, genotypes, and means (SD) for the nine evaluated traits

Trait	Number of animals	Number of animals with DFI	Number of phenotype records	Number of genotyped animals	Mean (SD)
DFI (kg)	4487		304,826	463	2.36 (0.60)
AGE (days)	74,365	4455	74,365	1555	135.10 (9.03)
BFT (mm)	73,967	4453	73,967	1552	7.63 (1.12)
LMT (mm)	73,935	4454	73,935	1541	71.50 (6.19)
FCR	12,379	4175	12,379	782	2.28 (0.20)
LMP (%)	37,810	1921	37,810	65	63.20 (2.04)
PH24	25,375	1577	25,375	34	5.68 (0.17)
DLP (%)	22,993	1301	22,993	29	0.03 (0.02)
BHW (kg)	18,325	668	18,325	16	9.98 (0.67)

DFI: Daily feed intake; AGE: the age at which the animal reached 100 kg; BFT: backfat thickness at 100 kg; LMT: loin muscle thickness at 100 kg; FCR: feed conversion ratio; LMP: lean meat percentage; PH24: pH of the ham at 24 h postmortem; DLP: drip loss percentage; BHW: boneless ham weight

The pedigree was extracted from the collective National French pig program and traced back from phenotypes for 10 generations, resulting in a total of 84,714 pigs in the pedigree. Among them, 1557 animals were genotyped (Table 1) with the porcine SNP60 Illumina BeadChip (Illumina, San Diego, CA). All the genotyped animals had phenotypes. Quality control of the genotypes was carried out using qcf90 [32]. Single nucleotide polymorphisms (SNPs) with call rates lower than 0.90, a minor allele frequency lower than 0.05, and deviating from Hardy–Weinberg equilibrium (*P* value < 0.05) were removed. After quality control, genotypes on 43,192 SNPs on 1549 animals were retained for analyses and used to construct the genomic relationship matrix.

### Estimation of probabilities of the occurrence of an unrecorded environmental challenge

The estimation of the probabilities of the occurrence of an unrecorded challenge is described in details in Tusingwiire et al. [26]. Briefly, the probabilities of occurrence of an unrecorded environmental challenge on a given day were estimated via a mixture model for the log CV of DFI for a total of 4741 days across all batches [26], using the method proposed by Garcia-Baccino et al. [9]. The CV of DFI were computed per batch to avoid the variability related to the fact that combining batches on the same day results in animals of varying ages across batches. The *normalmixEM* function from the R package *mixtools* [33] was used to fit the Gaussian mixture model via the Expectation–Maximization algorithm. To verify the presence of a mixture of two components, we applied a parametric bootstrap using the *emtest.norm* function from the *MixtureInf* R package [34]. The distribution of the log CV of DFI for all days was described in our previous study [26]. The CV of DFI across batches ranged from 0.10 to 1.31 and its distribution across days for nine randomly selected batches is presented in Additional file 1: Figure S1.

Days with a high probability of belonging to the first component of the mixture distribution (low variation) were "low CV days", whereas days with a high probability of belonging to the second component (high variation) were "high CV days". High CV days (stressful days) showed high variability in DFI and are most likely related to the occurrence of an unrecorded environmental challenge [9]. The probabilities $(p)$ of being “high CV days” were used in the genetic analysis as an environmental descriptor continuous covariable for DFI. In the following, when we indicate regression or slope on EG, we refer to the slope on the continuous covariable *p*. Since the other traits (AGE, BFT, LMT, FCR, LMP, PH24, DLP, and BHW) are measured once in the lifetime of the animal, it is not possible to estimate the probabilities of the occurrence of an unrecorded environmental challenge based on these traits.

### Variance component estimation

Variance components were estimated via the restricted maximum likelihood (REML) method [35] with the BLUPF90 + software [36, 37], available from https://nce.ads.uga.edu/wiki, using the ssGBLUP method, which integrates pedigree and genomic data into a unified evaluation [24, 25]. The ssGBLUP was fitted using a multivariate model with two observed phenotypes (DFI and one other trait, e.g. AGE) and three genetic effects: the intercept and slope of the reaction norm for DFI on the EG and one for the other trait. The reaction norm was modeled using the probability *p* of an unrecorded challenge as a continuous covariable (environmental descriptor), as described above. This approach allows us to capture the environmental sensitivity (slope of DFI) while analyzing its genetic correlations with the other economically important trait across the EG. It is not possible to fit the reaction norm on the other traits, because they were recorded only once per animal. The model can be described as follows:

$$\begin{aligned}\left[\begin{array}{c}{\mathbf{y}}_{1}\\ {\mathbf{y}}_{2}\end{array}\right]=\left[\begin{array}{c}{\mathbf{X}}_{1}\\ 0\end{array}\begin{array}{c}0\\ {\mathbf{X}}_{2}\end{array}\right]\left[\begin{array}{c}{{\varvec{\upbeta}}}_{1}\\ {{\varvec{\upbeta}}}_{2}\end{array}\right]+ \left[\begin{array}{c}{\mathbf{Z}}_{10}\\ 0\end{array}\begin{array}{c}{\mathbf{Z}}_{11}\\ 0\end{array}\begin{array}{c}0\\ {\mathbf{Z}}_{2}\end{array}\right]\left[\begin{array}{l}{\mathbf{a}}_{10}\\ {\mathbf{a}}_{11}\\ {\mathbf{a}}_{2}\end{array}\right]+ \\[6pt] \quad \left[\begin{array}{c}{\mathbf{W}}_{10}\\ 0\end{array}\begin{array}{c}{\mathbf{W}}_{11}\\ 0\end{array}\begin{array}{c}0\\ {\mathbf{W}}_{2}\end{array}\right]\left[\begin{array}{l}{\mathbf{p}\mathbf{e}}_{10}\\ {\mathbf{p}\mathbf{e}}_{11}\\ {\mathbf{p}\mathbf{e}}_{2}\end{array}\right]+\left[\begin{array}{c}{\mathbf{e}}_{1}\\ {\mathbf{e}}_{2}\end{array}\right],\end{aligned}$$

where ${\mathbf{y}}_{1}$ is the vector of DFI records (trait 1) (recorded daily) and ${\mathbf{y}}_{2}$ is the vector of observations for the other trait (trait 2) (recorded once per animal), ${\mathbf{X}}_{1}$ and ${\mathbf{X}}_{2}$ are the incidence matrices linking records to the fixed effects for DFI (${{\varvec{\upbeta}}}_{1}$) and the other trait (${{\varvec{\upbeta}}}_{2}$), ${\mathbf{Z}}_{10}$ and ${\mathbf{Z}}_{11}$ are incidence matrices linking records of DFI to the breeding values for the intercept (${\mathbf{a}}_{10}$) and the slope (${\mathbf{a}}_{11}$) for DFI, while ${\mathbf{Z}}_{2}$ is an incidence matrix relating the records with the breeding values for the other trait (${\mathbf{a}}_{2}$). The probability that, on a given day, an unrecorded environmental challenge occurred (*p*) is included in ${\mathbf{Z}}_{11}$ and is related to the slope of DFI, which quantifies the animal's environmental sensitivity across the EG. The incidence matrices ${\mathbf{W}}_{10}$ and ${\mathbf{W}}_{11}$ (which also includes the probability *p*) link records to the permanent environmental effects for the intercept (${\mathbf{p}\mathbf{e}}_{10}$) and the slope (${\mathbf{p}\mathbf{e}}_{11}$) for DFI, respectively; whereas ${\mathbf{W}}_{2}$ is the incidence matrix for the permanent environmental effect (${\mathbf{p}\mathbf{e}}_{2}$) for the other trait. The vectors ${\mathbf{e}}_{1}$ and ${\mathbf{e}}_{2}$ include the random residual effects for DFI and the other trait, respectively. The animal permanent environmental effect was modeled for both DFI and the other traits, although the latter only had a single record per animal, as it is assumed that the permanent environmental effects of DFI are correlated with those of single measured traits [38]. This considers the fact that, the residual covariance cannot be directly estimated, as the observations for DFI do not happen at the same time than the other trait, but still considers that effects linked to the animal other than additive genetic may result in similarity across traits. This approach ensures that all covariances across single measured traits and DFI are correctly modelled. The fixed effects included in the model for each trait are summarized in Table 2 and were selected based on prior biological knowledge, their use in national genetic evaluations, and statistical testing for their significance (P < 0.05) in the R software [39].

**Table 2:** Fixed effects included in the model for each trait

Trait	Fixed effects							
	Sex	Pen	Batch	Farm	Weight control	Hot carcass weight	Slaughter date	Sample weight
DFI	x	✓	✓	✓	x	x	x	x
AGE	✓	x	✓	✓	x	x	x	x
BFT	✓	x	✓	✓	✓	x	x	x
LMT	✓	x	✓	✓	✓	x	x	x
FCR	x	✓	✓	✓	✓	x	x	x
LMP	✓	x	✓	✓	x	✓	x	x
PH24	✓	x	✓	✓	x	✓	✓	x
DLP	✓	x	✓	✓	✓	x	✓	✓
BHW	✓	x	✓	✓	✓	x	x	x

DFI: Daily feed intake; AGE: the age at which the animal reached 100 kg; BFT: backfat thickness at 100 kg; LMT: loin muscle thickness at 100 kg; FCR: feed conversion ratio; LMP: lean meat percentage; PH24: pH of the ham at 24 h postmortem; DLP: drip loss percentage; BHW: boneless ham weight

The additive genetic variance was modelled according to $$\left[\begin{array}{l}{\mathbf{a}}_{10}\\ {\mathbf{a}}_{11}\\ {\mathbf{a}}_{2}\end{array}\right]\sim N\left(0,\mathbf{K}\otimes \mathbf{H}\right)$$, where $$\mathbf{K}=\left[\begin{array}{ccc}{\upsigma }_{{\text{a}}_{10}}^{2}& {\upsigma }_{{\text{a}}_{10}{\text{a}}_{11}}& {\upsigma }_{{\text{a}}_{10}{\text{a}}_{2}}\\ {\upsigma }_{{\text{a}}_{11}{\text{a}}_{10}}& {\upsigma }_{{\text{a}}_{11}}^{2}& {\upsigma }_{{\text{a}}_{11}{\text{a}}_{2}}\\ {\upsigma }_{{\text{a}}_{2}{\text{a}}_{10}}& {\upsigma }_{{\text{a}}_{2}{\text{a}}_{11}}& {\upsigma }_{{\text{a}}_{2}}^{2}\end{array}\right]$$, with ${\upsigma }_{{\text{a}}_{10}}^{2}$ the additive genetic variance of the intercept of DFI; ${\upsigma }_{{\text{a}}_{11}}^{2}$ the additive genetic variance of the slope of DFI on EG; ${\upsigma }_{{\text{a}}_{2}}^{2}$ the additive genetic variance for trait 2; and off-diagonals represent the corresponding genetic covariances. $\mathbf{H}$ is a combined relationship matrix that integrates the pedigree and genomic information [24, 25], and $\otimes$ is the Kronecker product. The permanent environmental variance was modeled as $$\left[\begin{array}{l}{\mathbf{p}\mathbf{e}}_{10}\\ {\mathbf{p}\mathbf{e}}_{11}\\ {\mathbf{p}\mathbf{e}}_{2}\end{array}\right]\sim N\left(0,\mathbf{T}\otimes \mathbf{I}\right)$$, where **T** = $$\left[\begin{array}{ccc}{\upsigma }_{{\text{pe}}_{10}}^{2}& {\upsigma }_{{\text{pe}}_{10}{\text{pe}}_{11}}& {\upsigma }_{{\text{pe}}_{10}{\text{pe}}_{2}}\\ {\upsigma }_{{\text{pe}}_{11}{\text{pe}}_{10}}& {\upsigma }_{{\text{pe}}_{11}}^{2}& {\upsigma }_{{\text{pe}}_{11}{\text{pe}}_{2}}\\ {\upsigma }_{{\text{pe}}_{2}{\text{pe}}_{10}}& {\upsigma }_{{\text{pe}}_{2}{\text{pe}}_{11}}& {\upsigma }_{{\text{pe}}_{2}}^{2}\end{array}\right]$$, with ${\upsigma }_{{\text{pe}}_{10}}^{2}$ the permanent environmental variance of the intercept of DFI; ${\upsigma }_{{\text{pe}}_{11}}^{2}$ the permanent environmental variance of the slope of DFI on EG; ${\upsigma }_{{\text{pe}}_{2}}^{2}$ the permanent environmental variance for trait 2; and off-diagonals represent the corresponding permanent environmental covariances. The residual variance was modeled as $$\left[\begin{array}{c}{\mathbf{e}}_{1}\\ {\mathbf{e}}_{2}\end{array}\right]\sim N(0,\mathbf{Q}\otimes \mathbf{I}$$), where $$\mathbf{Q}\hspace{0.17em}=\hspace{0.17em}\left[\begin{array}{c}{\upsigma }_{{\text{e}}_{1}}^{2}\\ 0\end{array}\begin{array}{c}0\\ {\upsigma }_{{\text{e}}_{2}}^{2}\end{array}\right]$$, with ${\upsigma }_{{\text{e}}_{1}}^{2}$ the residual variance of DFI; ${\upsigma }_{{\text{e}}_{2}}^{2}$ the residual variance of trait 2; and **I** the identity matrix.

For comparison, a series of multivariate ssGBLUP model with a regular Animal Model (AM), i.e. without including reaction norms, were fitted to estimate variance components. The RNAM was compared to the AM model for each pair of analyzed traits (DFI and any other traits), using a REML ratio test (REMLRT), which was calculated as ${X}^{2}=-2ln\left(likelihood for AM\right)+ 2ln\left(likelihood for RNAM\right)$, where ${X}^{2}$ is the chi-square statistic. The significance of the ${X}^{2}$ statistic was evaluated via a mixture of Chi-square distributions with 5 and 7 degrees of freedom [40, 41]. The null hypothesis assumes that the additive genetic and permanent environmental slope variances and slope-intercept covariances are zero. The mixtures of ${X}^{2}$ distributions arise because the parameters being tested are on the boundary of the parameter space, a property of likelihood ratio tests under non-standard conditions [40].

After obtaining the (co)variances components, the genetic correlation between DFI (${\text{a}}_{1}={\text{a}}_{10}+p {\text{a}}_{11}$) and the other trait [42] across the EG (probability of the occurrence of an environmental challenge on a given day, *p*) was estimated based on the estimated variance components of each character as: $\text{r}({a}_{1},{a}_{2})= \frac{{\text{Cov}(\text{a}}_{1},{\text{a}}_{2}) }{\sqrt{{\text{Var}(\text{a}}_{1}) {\text{Var}(\text{a}}_{2}) }}= \frac{{\text{Cov}(\text{a}}_{10} + {p\text{ a}}_{11}, {\text{ a}}_{2})}{\sqrt{{(\upsigma }_{{\text{a}}_{10}}^{2}+ {p}^{2}{\upsigma }_{{\text{a}}_{11}}^{2}+ {2p\upsigma }_{{\text{a}}_{10},{\text{a}}_{11}}){\upsigma }_{{\text{a}}_{2}}^{2}}}$. Note that $\text{r}({a}_{1},{a}_{2})=\text{r}({a}_{10},{a}_{2})$ when $p$=0. In order to obtain more information about the environmental sensitivity of the animals, we estimated the genetic correlation between the slope of DFI and the other trait as:$\text{r}\left({a}_{11},{a}_{2}\right)= \frac{\text{Cov}({\text{a}}_{11,}{\text{a}}_{2})}{\sqrt{{\upsigma }_{{\text{a}}_{11}}^{2} {\upsigma }_{{\text{a}}_{2}}^{2}}}.$

To assess how much DFI can be improved by genetic selection without altering environmental sensitivity, we also estimated the genetic correlation between the breeding value of DFI and the slope of DFI (sensitivity) across the EG as: $\text{r}\left({a}_{1},{a}_{11}\right)= \frac{\text{Cov}({\text{a}}_{10} + {p\text{ a}}_{11},{\text{a}}_{11})}{\sqrt{{(\upsigma }_{{\text{a}}_{10}}^{2}+ {p}^{2}{\upsigma }_{{\text{a}}_{11}}^{2}+ {2p\upsigma }_{{\text{a}}_{10},{\text{a}}_{11}}){\upsigma }_{{\text{a}}_{11}}^{2}}}$. Note that the intercept is truly insensitive to environments when this correlation is zero which strictly occurs at the value $p=-\frac{\text{Cov}({\text{a}}_{10}, {\text{a}}_{11})}{{\upsigma }_{{\text{a}}_{11}}^{2}}$ [43].

## Results

Goodness-of-fit of the RNAM and the AM was evaluated using the REMLRT (Table 3). The RNAM provided a significantly better fit of the data compared to AM for all trait pairs, as indicated by the P-values of the chi-square statistic. That is to say, the environmental covariate (the probability of the occurrence of a challenge on a given day) explained a significant proportion of the variation in DFI. These results justify the inclusion of the probability of the occurrence of an environmental challenge as an environmental descriptor in the genetic analysis.

**Table 3:** Restricted maximum likelihood ratio test (REMLRT) comparing bivariate models for daily feed intake (DFI) and other production traits with (RNAM) and without (AM) inclusion of an environmental gradient for DFI

Traits	− 2 Log-Likelihood		LRT	
	AM	RNAM	χ^2^	P-value
DFI-AGE	881,941.77	868,811.53	13,130.24	p < 0.0001
DFI-BFT	559,758.28	546,479.09	13,279.19	p < 0.0001
DFI-LMT	796,114.77	782,843.03	13,271.74	p < 0.0001
DFI-FCR	356,623.06	343,434.96	13,188.10	p < 0.0001
DFI-LMP	524,503.18	511,215.86	13,287.32	p < 0.0001
DFI-PH24	346,118.14	332,845.72	13,272.42	p < 0.0001
DFI-DLP	455,067.78	441,798.60	13,269.18	p < 0.0001
DFI-BHW	384,808.03	371,538.62	13,269.41	p < 0.0001

RNAM: reaction norm animal model; AM: regular animal model

${\chi }^{2}$: chi-square value

AGE: the age at which the animal reached 100 kg; BFT: backfat thickness at 100 kg; LMT: loin muscle thickness at 100 kg; FCR: feed conversion ratio; LMP: lean meat percentage; PH24: pH of the ham at 24 h postmortem; DLP: drip loss percentage; BHW: boneless ham weight

Genetic parameter estimates between DFI and the other traits (AGE, BFT, LMT, FCR, LMP, PH24, DLP, and BHW) analyzed using the RNAM model are presented in Table 4. The estimates from RNAM at *p* = 0 were similar to those from AM (see Additional file 2: Table S1).

**Table 4:** Genetic parameter estimates between daily feed intake (DFI) and production traits analyzed using a reaction norm animal model for DFI. DFI is denoted by subscript 1 and the other traits use the subscript 2 in each analysis

Traits	${\sigma }_{{a}_{10}}^{2}$	${\sigma }_{{a}_{11}}^{2}$	${\sigma }_{{a}_{2}}^{2}$	${\sigma }_{{a}_{10}{a}_{11}}$	${\sigma }_{{a}_{10}{a}_{2}}$	${\sigma }_{{a}_{11}{a}_{2}}$	${\sigma }_{{pe}_{10}}^{2}$	${\sigma }_{{pe}_{11}}^{2}$	${\sigma }_{{pe}_{2}}^{2}$	${\sigma }_{{pe}_{10}{pe}_{11}}$	${\sigma }_{{pe}_{10}{pe}_{2}}$	${\sigma }_{{pe}_{11}{pe}_{2}}$	${\sigma }_{{e}_{1}}^{2}$	${\sigma }_{{e}_{2}}^{2}$	${r}_{{a}_{10}{a}_{2}}$	${r}_{{a}_{11}{a}_{2}}$
AGE	0.036	0.087	34.773	− 0.021	− 0.918	0.254	0.030	0.122	30.422	− 0.004	− 0.836	− 0.776	0.175	12.420	− 0.817	0.146
BFT	0.037	0.089	0.483	− 0.013	0.063	0.007	0.024	0.125	0.127	− 0.016	0.009	0.003	0.175	0.381	0.470	0.035
LMT	0.033	0.093	10.389	− 0.018	0.048	− 0.282	0.026	0.123	5.662	− 0.014	− 0.027	0.206	0.175	7.708	0.081	− 0.286
FCR	0.043	0.088	0.018	− 0.013	0.025	0.002	0.023	0.122	0.004	− 0.015	0.009	0.002	0.175	0.009	0.901	0.052
LMP	0.034	0.089	1.329	− 0.016	− 0.068	− 0.024	0.025	0.124	0.945	− 0.014	− 0.064	0.081	0.175	1.878	− 0.318	− 0.069
PH24	0.033	0.089	0.006	− 0.017	0.003	− 0.003	0.026	0.125	0.006	− 0.014	0.001	0.003	0.175	0.012	0.177	− 0.130
DLP	0.033	0.089	1.143	− 0.017	− 0.042	0.019	0.026	0.125	1.846	− 0.014	− 0.012	0.002	0.175	0.0003	− 0.214	0.059
BHW	0.034	0.089	0.040	− 0.017	− 0.012	0.009	0.026	0.124	0.017	− 0.014	0.006	− 0.007	0.175	0.097	− 0.315	0.149

${\sigma }_{{a}_{10}}^{2}$: additive genetic variance of the intercept of DFI; ${\sigma }_{{a}_{11}}^{2}$: additive genetic variance of the slope of DFI; ${\sigma }_{{a}_{2}}^{2}$: additive genetic variance of the second trait; ${\sigma }_{{a}_{10}{a}_{11}}$:covariance between the intercept and the slope of DFI; ${\sigma }_{{a}_{10}{a}_{2}}$: covariance between the intercept of DFI and the second trait; ${\sigma }_{{a}_{11}{a}_{2}}$: covariance between the slope of DFI and the second trait; ${\sigma }_{{pe}_{10}}^{2}$: variance of the permanent environmental effects of the intercept of DFI; ${\sigma }_{{pe}_{11}}^{2}$: variance of the permanent environmental effects of the slope of DFI; ${\sigma }_{{pe}_{2}}^{2}$: variance of the permanent environmental effects of the second trait; ${\sigma }_{{pe}_{10}{pe}_{11}}$: covariance between intercept and slope of DFI of the permanent environmental effects; ${\sigma }_{{pe}_{10}{pe}_{2}}$: covariance between the permanent environmental effects of intercept of DFI and the second trait; ${\sigma }_{{pe}_{11}{pe}_{2}}$: covariance between the permanent environmental effects of slope of DFI and the second trait; ${\sigma }_{{e}_{1}}^{2}$: residual variance of DFI; ${\sigma }_{{e}_{2}}^{2}$: residual variance the second trait; and ${r}_{{a}_{10}{a}_{2}}$= genetic correlation between DFI at p = 0 and the second trait; {r}_{{a}_{11}{a}_{2}}= genetic correlation of the slope DFI with the second trait.

AGE: the age at which the animal reached 100 kg; BFT: backfat thickness at 100 kg; LMT: loin muscle thickness at 100 kg; FCR: feed conversion ratio; LMP: lean meat percentage; PH24: pH of the ham at 24 h postmortem; DLP: drip loss percentage; BHW: boneless ham weight

**Fig. 1:**
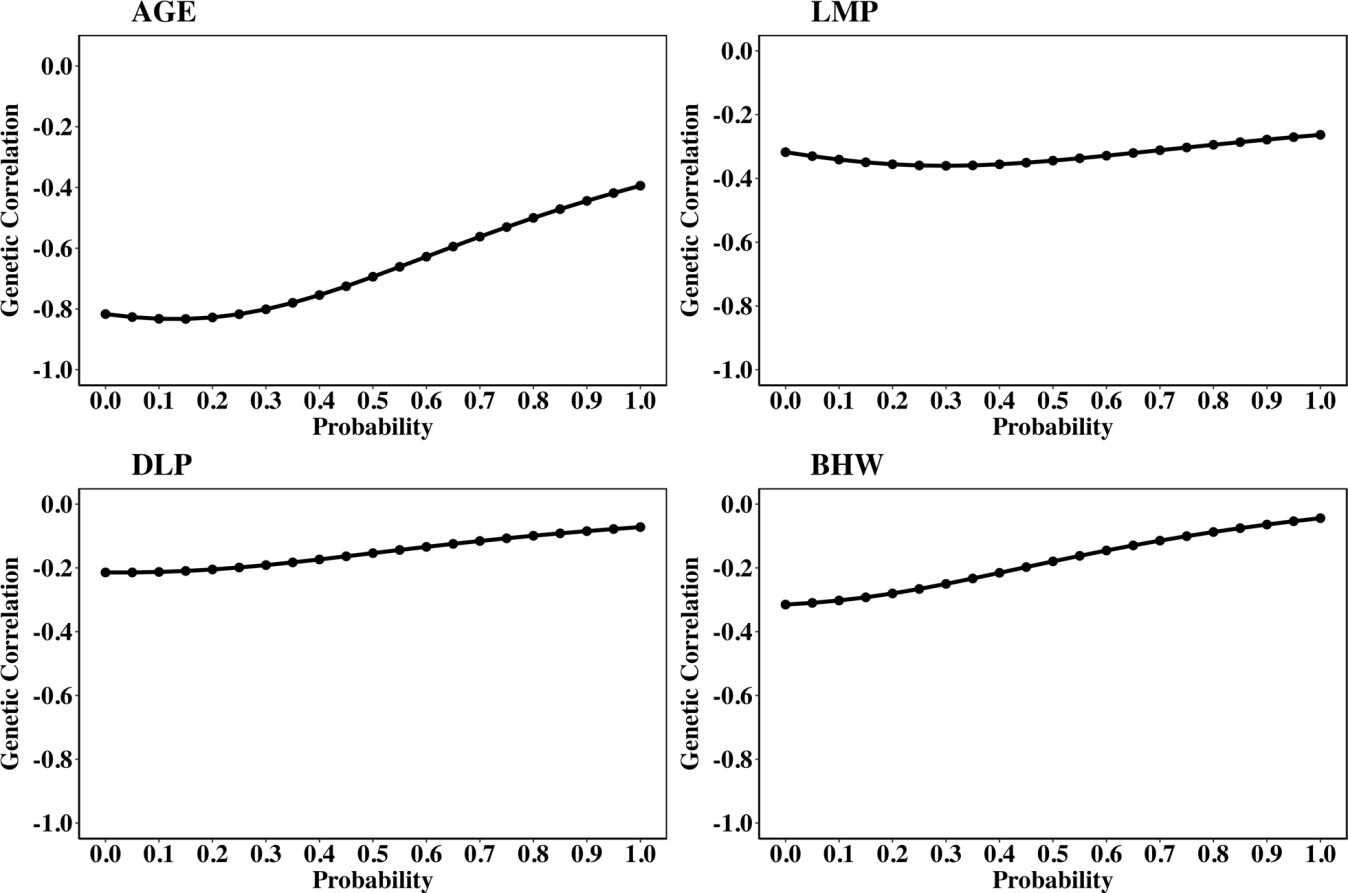
Estimates of genetic correlations of daily feed intake (DFI) with AGE, LMP, DLP, and BHW across the environmental gradient. AGE: the age at which the animal reached 100 kg; LMP: lean meat percentage; DLP: drip loss percentage; BHW: boneless ham weight. The probability (*p*) is the environmental descriptor; *p* = 0 indicates non-challenging environmental conditions, while *p* = 1 indicates highly challenging conditions

Estimates of genetic correlations between DFI and AGE, LMP, DLP, and BHW increased across the EG, as shown in Fig. 1. The genetic correlations from *p* = 0 (non-stressful environment) to *p* = 1 (stressful environment) ranged from – 0.82 to – 0.39 for DFI and AGE, from − 0.32 to − 0.26 for DFI and LMP, from – 0.21 to – 0.07 for DFI and DLP, and from – 0.31 to – 0.04 for DFI and BHW. Thus, the increase in genetic correlation estimates across the EG (from *p* = 0 to *p* = 1) of DFI with the other traits was 0.43, 0.06, 0.14, and 0.27 for AGE, LMP, DLP, and BHW, respectively.

**Fig. 2:**
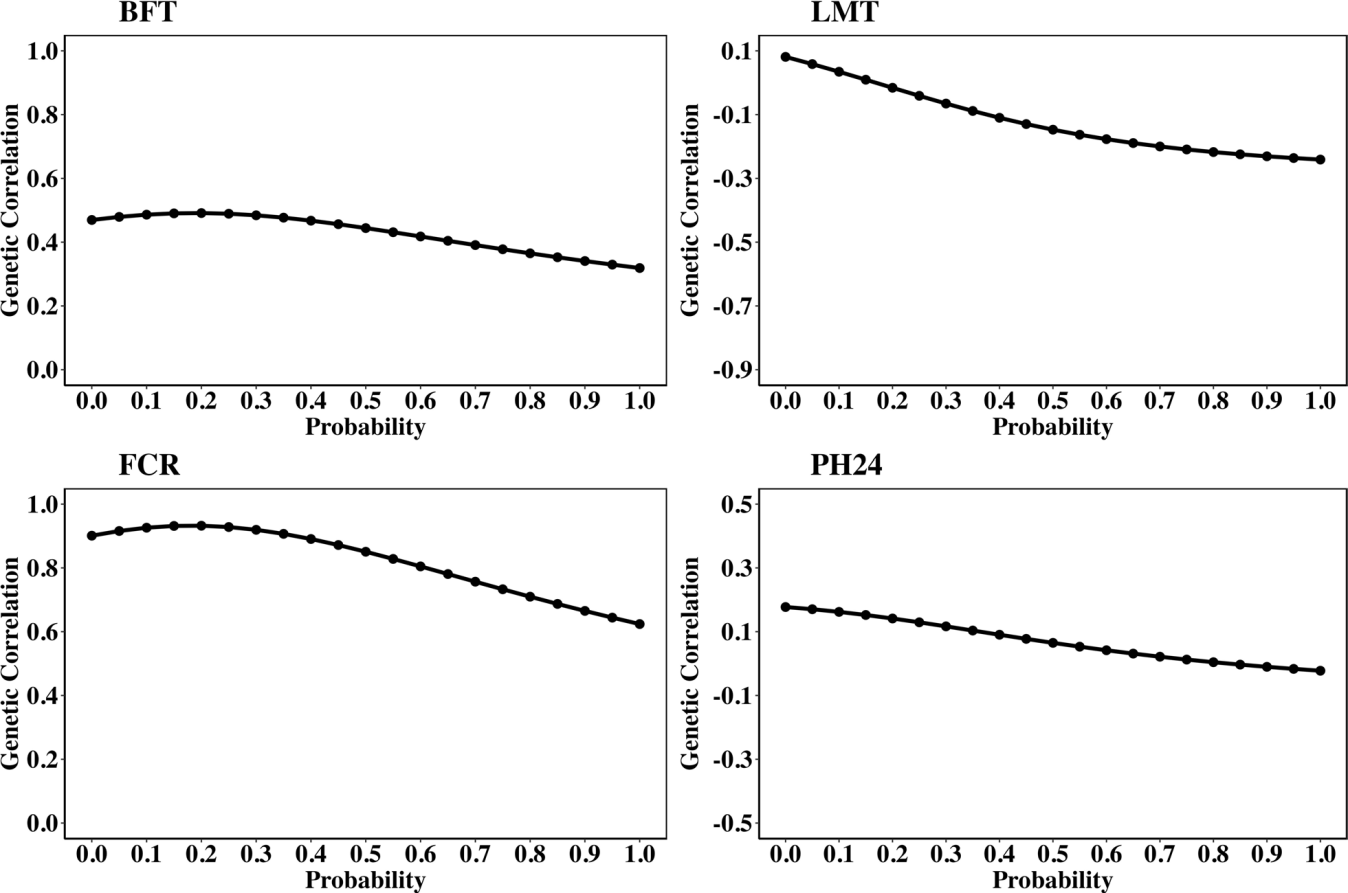
Estimates of genetic correlations of daily feed intake (DFI) with BFT, LMT, FCR, and PH24 across the environmental gradient. BFT: backfat thickness at 100 kg; LMT: loin muscle thickness at 100 kg; FCR: feed conversion ratio; PH24: pH of the ham at 24 h postmortem. The probability (*p*) is the environmental descriptor; *p* = 0 indicates non-challenging environmental conditions, while *p* = 1 indicates highly challenging conditions

Estimates of genetic correlations of DFI with BFT, LMT, FCR, and PH24 across the EG (from *p* = 0 to *p* = 1) decreased, as shown in Fig. 2. Estimates of the genetic correlation (from *p* = 0 to *p* = 1) ranged from 0.47 to 0.32 for DFI and BFT, from 0.08 to – 0.24 for DFI and LMT, from 0.90 to 0.62 for DFI and FCR, and from 0.18 to -0.02 for DFI and PH24. The decrease of genetic correlation estimates of DFI with the other traits from non-stressful (*p* = 0) to stressful conditions (*p* = 1) was – 0.15, – 0.32, – 0.28, and – 0.20 for BFT, LMT, FCR, and PH24, respectively.

Estimates of the genetic correlation of the slope of DFI on EG with production traits were 0.15 for AGE, 0.04 for BFT, – 0.29 for LMT, 0.05 for FCR, – 0.07 for LMP, – 0.13 for PH24, 0.06 for DLP, and 0.15 for BHW (Table 4). Overall, estimates of the genetic correlation of the environmental sensitivity of the animals (the slope of DFI on EG) with the other production traits were low, except for LMT, which showed a moderate genetic correlation. This indicates that selection for resilience based on the environmental sensitivity (slope of DFI) can be performed without adversely affecting these other production traits.

**Fig. 3:**
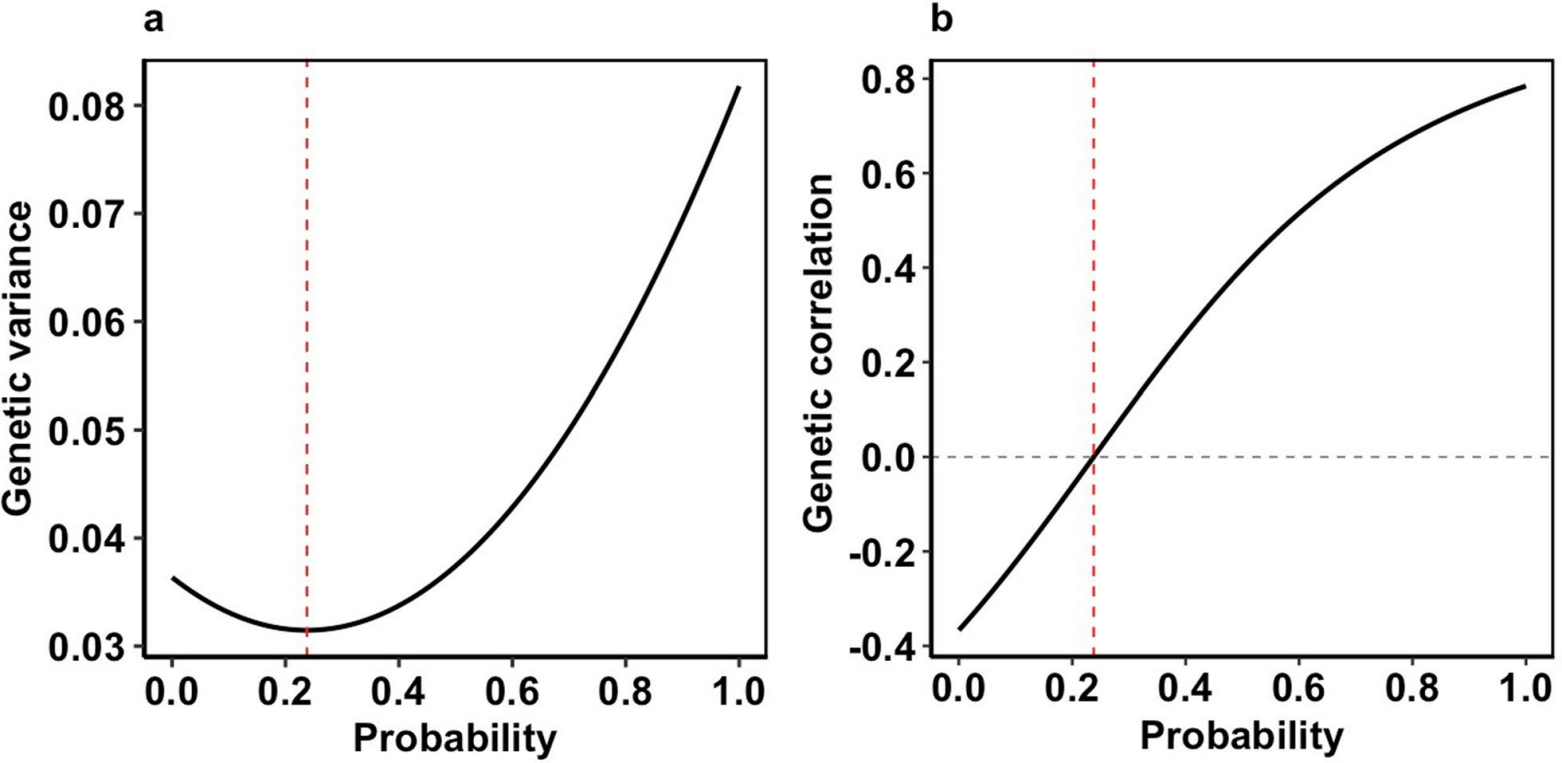
Estimates of the total additive genetic variance of daily feed intake (DFI) (**a**) and of the genetic correlation between the total breeding value of DFI and the slope of DFI (**b**) across the probabilities of the occurrence of a challenge. The probability (*p*) is the environmental descriptor; *p* = 0 indicates non-challenging environmental conditions, while *p* = 1 indicates highly challenging conditions. The red dotted line indicates the point where the genetic correlation between the intercept and the slope of DFI would be zero

The analyses provided three genetic correlations related to DFI. The estimate of the genetic correlation between the intercept and the slope of DFI, equal to – 0.35, was reported in our previous work (Table 5 in [26]). Estimates of the genetic correlation of the intercept and the total breeding value for DFI along EG is presented in Fig. 5 in [26] and ranged from 1 to 0.2. Here, we estimated the genetic correlation between the slope of DFI on EG (environmental sensitivity) and the total breeding value for DFI (${\text{a}}_{1}={\text{a}}_{10}+p {\text{a}}_{11}$) along the EG (Fig. 3). Figure 3a shows the estimates of total additive genetic variance for DFI for increasing probabilities of the occurrence of an environmental challenge, which ranged from 0.036 for *p* = 0 (non-challenging environment) to 0.082 for *p* = 1 (challenging environment). The genetic variance initially decreased slightly due to the negative genetic covariance between the intercept and the slope of DFI (− 0.018 in Tusingwiire et al. [26]) and then increased at higher values of the probability of a challenge. The point along the EG where the genetic correlation between the intercept and slope of DFI was estimated to be zero was at *p* = 0.24 (indicated by the red dotted line in Fig. 3). At this point, the genetic variance of DFI was 0.031, the lowest along the gradient. Figure 3b also shows estimates of the genetic correlation between the slope of DFI (sensitivity) and DFI across the EG, which ranged from – 0.37 at *p* = 0 to 0.78 at *p* = 1. At *p* = 0.24, the intercept was estimated to be independent of the environmental sensitivity (slope of DFI) and therefore selection at this point is not expected to change the slope of DFI [44].

## Discussion

Here, we primarily investigated the genetic correlations of the slope of DFI on EG, as an indicator of animals’ sensitivity to environmental challenges with other traits of economic importance for Piétrain pigs using a single-step RNAM. Differences in real world environmental conditions were described by the estimated probability of a high CV of DFI on a given day, i.e. the probability of a challenge occurring. In addition, we estimated the genetic correlation between DFI (its total breeding value) and the same traits across the EG. Note that analysis of the DFI on its own does not tell us anything about the sensitivity of the animals.

There is limited literature exploring the genetic correlation of the slope of DFI on EG as a proxy of environmental sensitivity with other economically important traits in pigs. Notably, Schnyder et al. [38] estimated the genetic correlation of the linear regression coefficient of the slope of DFI with production traits in Large White pigs. They reported genetic correlations of 0.38, 0.48, – 0.55, and 0.57 of the slopes of DFI with average daily gain, feed conversion ratio, carcass lean content, and meat quality index, respectively. Similarly, Eissen [44] reported comparable estimates of genetic correlations of the slope of the linear regression coefficient of the DFI on test days with other performance traits in Duroc pigs. In the current study, the genetic correlation of the slope of DFI on EG with other production traits were lower than those reported by Eissen [44] and Schnyder et al. [38]. Eissen [44] noted that animals with steeper slopes of DFI on test days tend to deposit more fat, as reflected by the positive genetic correlation between the slope of DFI and BFT (0.50) and a negative genetic correlation between the slope of DFI and carcass lean content (– 0.61). In our study, we reported a moderately negative genetic correlation between the slope of DFI and LMT (– 0.29), indicating that animals with higher breeding values for environmental sensitivity tend to have reduced muscle development. The remaining traits exhibited weak genetic correlation estimates with the slope of DFI, suggesting that selection for resilience based on the slope of DFI (environmental sensitivity) would have a minimal effect on these other traits.

Estimates of genetic correlations between DFI and other traits varied across the EG (from *p* = 0 to *p* = 1) (Figs. 1 and 2). The high negative genetic correlation between DFI and AGE under non-stressful environmental conditions (– 0.82 at *p* = 0) suggests that pigs with higher breeding values for DFI at this level exhibit faster growth rate and reach market weight earlier (have less AGE). These animals have the capacity to adjust their feed intake significantly in response to favorable conditions, which translates into accelerated growth, a trait that is highly desired and economically important in pig production. This is in line with the fact that selection has been primarily based on performance under ideal conditions in nucleus farms for many generations. The genetic correlation between DFI and AGE was estimated to become less negative as the environmental conditions become more challenging (from – 0.82 at *p* = 0 to – 0.39 at *p* = 1). These genetic correlation estimates are higher than the values reported in literature between average DFI across the test period and AGE (– 0.36 for Yorkshire pigs and – 0.25 for Duroc pigs) [45, 46]. Moreover, DFI is positively correlated with average daily gain, as reported Nuñez et al. [47] (0.68 for Piétrain), Do et al. [48] (0.45 for Duroc, 0.72 for Landrace, and 0.84 for Yorkshire), Jiao et al. [49] (0.32 for Duroc), and Kavlak et al. [50] (0.67 to 0.86 across the growth period in Yorkshire), indicating that higher DFI promotes growth. The genetic correlations between DFI and AGE becoming less negative in stressful conditions indicates that in such environments, animals take longer to reach market weight (i.e., have higher AGE) than in nonstressful environments. These results suggest that the efficiency of pigs in utilizing feed for growth is compromised under stress, potentially due to resource allocation trade-offs. In stressful conditions, animals may allocate more resources to maintenance or survival response rather than growth, resulting in slower growth. It is important to note that although the estimate of the genetic correlation between the slope of DFI and AGE was low (0.15), this was sufficient to alter the estimate of the genetic correlation between DFI and AGE along the EG. This suggests that even low environmental sensitivity for DFI can lead to the re-ranking of animal based on breeding values of AGE across varying levels of environmental challenge. Challenging environmental conditions [1, 36, 37] such as heat stress increase respiration rates in pigs due to their inability to sweat, leading to panting for thermoregulation, which diverts energy away from growth [51]. Moreover, stress weakens the immune system, increasing susceptibility to infectious diseases, which further reduces growth [1, 2]. Not being able to adapt to challenging conditions, negatively affects feed efficiency and increases time (AGE) to reach market weight. Therefore, selection strategies that improve the growth rate in non-stressful environments may not yield the same results in more stressful conditions, making it crucial to consider environmental sensitivity in breeding programs to ensure consistent performance across different environments.

The highly positive genetic correlation (0.90) between DFI and FCR in non-stressful conditions estimated in our study suggests that animals with higher breeding values for DFI are less feed efficient (higher FCR), while those with lower breeding values for DFI exhibit higher feed efficiency (lower FCR). These results also suggest that selecting for animals with lower DFI can lead to improved feed efficiency (lower FCR). However, the estimate of the genetic correlation between DFI and FCR decreased (from 0.90 at *p* = 0 to 0.62 at *p* = 1) as the environmental conditions became more stressful. Despite the low genetic correlation estimate between the slope of DFI and FCR (0.05), this value was sufficient to alter the estimate of the genetic correlation between DFI and FCR along the EG, indicating that the genetics of feed efficiency is highly responsive to environmental variations. This implies that genetic selection to improve feed efficiency in non-stressful conditions might not always lead to the same improvements in feed efficiency in stressful environments. Our estimates of the genetic correlation obtained between DFI and FCR across the EG are comparable to those reported in the literature in different rearing conditions. Do et al. [48] reported genetic correlations between DFI and FCR of 0.74, 0.67, and 0.43 for Duroc, Danish Landrace, and Yorkshire, respectively. Bouquet et al. [52] also estimated a genetic correlation of 0.69 between the same traits in Large White, while Núñez et al. [47] estimated a genetic correlation of 0.62 in Piétrain pigs.

Our estimates of the genetic correlations of DFI with carcass composition traits in non-stressful conditions (0.47, 0.08, – 0.32, – 0.31 for BFT, LMT, LMP, and BHW, respectively) indicates that animals with higher breeding values for DFI tend to have higher BFT (as indicated by the positive genetic correlation between DFI and BFT), and lower muscle-related traits (as evidenced by the negative correlations between DFI and both LMP and BHW). However, the reduction in genetic correlation estimates between DFI and BFT (from 0.47 at *p* = 0 to 0.32 at *p* = 1), together with the shift in the genetic correlation between DFI and LMT from positive (0.08 at p = 0) to negative (–0.24 at p = 1) as conditions become more stressful, suggests that animals with higher breeding values for DFI tend to deposit less fat and muscle, under stressful conditions. This is because, under stressful conditions animals prioritize energy for maintenance and survival over growth, while also experiencing compromised immune function [1, 51, 53–55]. Moreover, the estimate of the genetic correlation of the slope of DFI with LMT was – 0.29, indicating that genetically more sensitive animals (i.e., animals with steeper slopes) will tend to have less muscle development. Therefore, selecting animals with flatter DFI slopes can enhance the stability of carcass composition traits, ensuring consistent performance across both favorable and challenging environments.

The low genetic correlation estimates of DFI with meat quality traits from non-stressful conditions to stressful conditions (– 0.21 to – 0.07 for DLP and 0.18 to – 0.02 for PH24) suggest that DFI has a limited effect on these traits. The fact that the genetic correlation estimates of the slope of DFI with these traits was low (– 0.13 for PH24 and 0.06 DLP) indicates that selection for reduced environmental sensitivity is not expected to have a significant effect on meat quality traits. Estimates of the genetic correlation between DFI and meat quality traits along the EG reported in this study are lower than those reported in the literature [56]. Déru [56] reported moderate to strong negative genetic correlations between DFI and PH24, with estimates of 0.31 for pigs on a conventional diet and 0.54 for those fed a high-fiber diet.

Precision livestock farming technologies are transforming modern animal breeding by facilitating the collection of detailed real-time longitudinal data through advanced sensors and computer visions. For example, automated feeding stations are used to measure DFI and monitor changes in feeding behavior under varying environmental conditions [7, 57]. In this study, we showed that genetic correlations between DFI collected from automated feeding stations and other traits of economic importance change across the EG, which suggests the presence of genotype-by-environment interactions (GxE). Ignoring GxE in genetic evaluations may result in a reduced selection response if selection is performed in a different environment than in which the commercial animals are performing [58]. In other words, animals may perform differently in specific environments due to variations in how their genetic potential is expressed under different environmental conditions. This is even more important as genetic selection in pig breeding programs is predominantly conducted within nucleus farms, where environmental conditions such as climate, health status, nutrition, and management practices differ markedly from those encountered in commercial production systems [18]. This could lead to a re-ranking of selection candidates based on EBVs across different environments. As a result, animals selected based on their EBVs in one specific environment may not exhibit optimal performance under contrasting environmental conditions. Thus, when G × E is present, breeding programs may need to be modified to account for the differences in genetic expression across different environments [59] to ensure that animals perform consistently across challenging environments.

In this study, differences in the variation of DFI on a given day within a batch of pigs (high CV of DFI) were assumed to be caused by the occurrence of a challenge on a given day. Note that we used data from nucleus breeding farms, which have a standardized management. However, it is important to acknowledge that variation in feeding patterns exists between and within pigs irrespective of challenges. For example, the CV may capture variability arising from factors unrelated to stress, such as natural developmental processes. As pigs mature, the variability in feed intake often decreases, reflecting physiological growth patterns rather than external challenges. Birth weight also influences the variation in feed intake at later growth stages [60]. Nevertheless, DFI can be easily measured using automatic feeding stations, reflecting day-to-day metabolic dynamics, and if only production data are available, variation in DFI within and between individuals may serve as a proxy for the animal’s sensitivity to environmental challenges [61]. During the growing period, animals are exposed to a wide range of changing environmental factors, including microclimate, nutrition, health status, social interactions, and internal physiological demands [61]. Animals with high environmental sensitivity respond strongly to these changes in the environment, as metabolic thresholds may be exceeded in sensitive individuals but remain uncompromised in more resilient animals [61]. Since animals differ in their metabolic threshold levels (e.g. metabolic heat production), their corrective metabolic responses (e.g. reduced metabolic rate, physical activity) also vary, resulting in variations in DFI (energy intake) [51, 61]. Resilience can be improved by selecting on the slope of DFI, with flatter slopes indicating less sensitivity to environmental challenges [9, 26].

## Conclusions

Estimates of genetic correlations of DFI with other traits of economic importance varied across the EG, which suggests the presence of GxE. Most of the traits of economic importance showed weak genetic correlations with the slope of DFI (an indicator of sensitivity of the animals to environmental challenges). Selecting animals for resilience based on environmental sensitivity (slope of DFI) is possible without negatively affecting production traits. Our results are useful for pig breeders, as they show the feasibility of improving resilience through genetic selection.

## Supplementary Information

Additional file 1. Figure S1. The distribution of the coefficient of variation (CV) of DFI across the days for 9 randomly selected batches.

Additional file 2. Table S1. Genetic parameter estimates between daily feed intake (DFI) and other traits analyzed using an animal model. DFI is denoted by subscript 1 and the other traits are denoted by subscript 2 in each analysis. ${\sigma }_{{a}_{1}}^{2}$: additive genetic variance of DFI; ${\sigma }_{{a}_{2}}^{2}$: additive genetic variance of the second trait; ${\sigma }_{{a}_{1}{a}_{2}}$: covariance between DFI and the second trait; ${\sigma }_{{pe}_{1}}^{2}$: variance of the permanent environmental effects of DFI; ${\sigma }_{{pe}_{1}}^{2}$:variance of the permanent environmental effects of the second trait;, ${\sigma }_{{pe}_{1}{pe}_{2}}$: covariance between the permanent environmental effects of DFI and the second trait; ${\sigma }_{{e}_{1}}^{2}$: residual variance of DFI; ${\sigma }_{{e}_{2}}^{2}$: residual variance the second trait; and ${r}_{{a}_{1}{a}_{2}}$= genetic correlation between DFI and the second trait. AGE: the age at which the animal reached 100 kg; BFT: backfat thickness at 100 kg; LMT: loin muscle thickness at 100 kg; FCR: feed conversion ratio; LMP: lean meat percentage; PH24: pH of the ham at 24 h postmortem; DLP: drip loss percentage; BHW: boneless ham weight.

## Data Availability

The data that support the findings of this study are available from SAS NUCLEUS, but restrictions apply to the availability of these data, which are not publicly available. Software is available at https://nce.ads.uga.edu/wiki.
