# Supplementary figures and images for: Genetic correlations of environmental sensitivity based on daily feed intake perturbations with economically important traits in a male pig line
Source: Genet Sel Evol. 2025 Oct 2;57:54. doi: 10.1186/s12711-025-01000-1 (PMC12492946; doi:10.1186/s12711-025-01000-1)

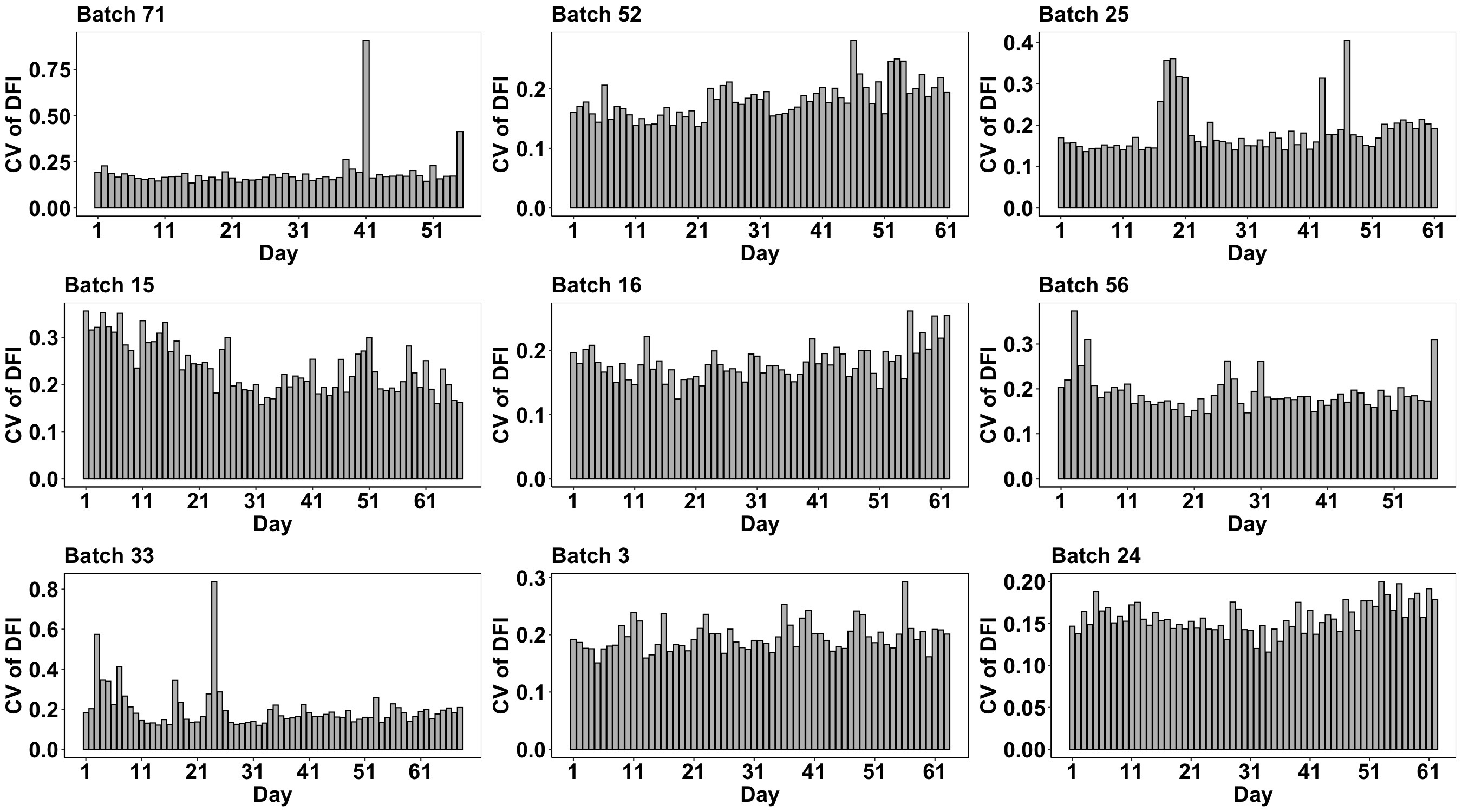

Supplement: Supplementary file 1 — Additional file 1. Figure S1. The distribution of the coefficient of variation (CV) of DFI across the days for 9 randomly selected batches. [file 12711_2025_1000_MOESM1_ESM.jpeg]
