# Supplementary material for: Genetic correlations of environmental sensitivity based on daily feed intake perturbations with economically important traits in a male pig line
Source: Genet Sel Evol. 2025 Oct 2;57:54. doi: 10.1186/s12711-025-01000-1 (PMC12492946; doi:10.1186/s12711-025-01000-1)
Supplement: Supplementary file 2 — Additional file 2. Table S1. Genetic parameter estimates between daily feed intake (DFI) and other traits analyzed using an animal model. DFI is denoted by subscript 1 and the other traits are denoted by subscript 2 in each analysis. ${\sigma }_{{a}_{1}}^{2}$σa12: additive genetic variance of DFI; ${\sigma }_{{a}_{2}}^{2}$σa22: additive genetic variance of the second trait; ${\sigma }_{{a}_{1}{a}_{2}}$σa1a2: covariance between DFI and the second trait; ${\sigma }_{{pe}_{1}}^{2}$σpe12: variance of the permanent environmental effects of DFI; ${\sigma }_{{pe}_{1}}^{2}$σpe12:variance of the permanent environmental effects of the second trait;, [file 12711_2025_1000_MOESM2_ESM.docx]

**Table S1 Genetic parameter estimates between daily feed intake (DFI) and other traits analyzed using an animal model. DFI is denoted by subscript 1 and the other traits use the subscript 2 in each analysis**.

| **Traits** | $\sigma_{a_{1}}^{2}$ | $\sigma_{a_{\boldsymbol{2}}}^{2}$ | $\sigma_{a_{1}a_{2}}$ | $\sigma_{\boldsymbol{pe}_{1}}^{2}$ | $\sigma_{\boldsymbol{pe}_{\boldsymbol{2}}}^{2}$ | $\sigma_{\boldsymbol{pe}_{1}\boldsymbol{pe}_{2}}$ | $\sigma_{e_{\boldsymbol{1}}}^{2}$ | $\sigma_{e_{\boldsymbol{2}}}^{2}$ | $r_{a_{1}a_{2}}$ | h^2^ |
| --- | --- | --- | --- | --- | --- | --- | --- | --- | --- | --- |
| AGE | 0.032 | 34.687 | -0.868 | 0.035 | 29.844 | -1.019 | 0.187 | 13.030 | -0.829 ± 0.02 | 0.447±0.01 |
| BFT | 0.037 | 0.482 | 0.062 | 0.023 | 0.119 | 0.008 | 0.187 | 0.389 | 0.460 ± 0.05 | 0.436±0.01 |
| LMT | 0.031 | 10.342 | 0.014 | 0.026 | 6.295 | -0.001 | 0.187 | 7.100 | 0.025 ± 0.06 | 0.436±0.01 |
| FCR | 0.042 | 0.018 | 0.026 | 0.024 | 0.011 | 0.011 | 0.187 | 0.003 | 0.930 ± 0.003 | 0.566±0.01 |
| LMP | 0.032 | 1.328 | -0.073 | 0.025 | 0.591 | -0.048 | 0.187 | 2.232 | -0.352 ± 0.07 | 0.320±0.01 |
| PH24 | 0.031 | 0.006 | 0.002 | 0.025 | 0.0001 | 0.001 | 0.187 | 0.017 | 0.148 ± 0.08 | 0.264±0.02 |
| DLP | 0.031 | 1.143 | -0.041 | 0.025 | 0.973 | -0.011 | 0.187 | 0.873 | -0.215±0.08 | 0.382±0.01 |
| BHW | 0.032 | 0.040 | -0.011 | 0.025 | 0.009 | 0.005 | 0.187 | 0.011 | -0.300± 0.10 | 0.261±0.02 |

$\sigma_{a_{1}}^{2}$: additive genetic variance of DFI; $\sigma_{a_{\mathbf{2}}}^{2}$: additive genetic variance of the second trait; $\sigma_{a_{1}a_{2}}$: covariance between DFI and the second trait; $\sigma_{\mathrm{pe}_{1}}^{2}$: variance of the permanent environmental effects of DFI; $\sigma_{\mathrm{pe}_{1}}^{2}$: variance of the permanent environmental effects of the second trait; , $\sigma_{\mathrm{pe}_{1}\mathrm{pe}_{2}}$: covariance between the permanent environmental effects of DFI and the second trait; $\sigma_{e_{\mathbf{1}}}^{2}$: residual variance of DFI; $\sigma_{e_{\mathbf{2}}}^{2}$: residual variance the second trait; and $r_{a_{1}a_{2}}$: genetic correlation between DFI and the second trait; h^2^: heritability of the second trait

AGE: the age at which the animal reached 100 kg; BFT: backfat thickness at 100 kg; LMT: loin muscle thickness at 100 kg; FCR: feed conversion ratio; LMP: lean meat percentage; PH24: pH of the ham at 24 hours postmortem; DLP: drip loss percentage; BHW: boneless ham weight.
